# Arsenic Exposure and Prevalence of the Varicella Zoster Virus in the United States: NHANES (2003–2004 and 2009–2010)

**DOI:** 10.1289/ehp.1408731

**Published:** 2015-01-30

**Authors:** Andres Cardenas, Ellen Smit, E. Andres Houseman, Nancy I. Kerkvliet, Jeffrey W. Bethel, Molly L. Kile

**Affiliations:** 1 School of Biological and Population Health Sciences, College of Public Health and Human Sciences, and; 2 Department of Environmental and Molecular Toxicology, College of Agricultural Sciences, Oregon State University, Corvallis, Oregon, USA

## Abstract

**Background:**

Arsenic is an immunotoxicant. Clinical reports observe the reactivation of varicella zoster virus (VZV) in people who have recovered from arsenic poisoning and in patients with acute promyelocytic leukemia that have been treated with arsenic trioxide.

**Objective:**

We evaluated the association between arsenic and the seroprevalence of VZV IgG antibody in a representative sample of the U.S. population.

**Methods:**

We analyzed data from 3,348 participants of the National Health and Nutrition Examination Survey (NHANES) 2003–2004 and 2009–2010 pooled survey cycles. Participants were eligible if they were 6–49 years of age with information on both VZV IgG and urinary arsenic concentrations. We used two measures of total urinary arsenic (TUA): TUA1 was defined as the sum of arsenite, arsenate, monomethylarsonic acid, and dimethylarsinic acid, and TUA2 was defined as total urinary arsenic minus arsenobetaine and arsenocholine.

**Results:**

The overall weighted seronegative prevalence of VZV was 2.2% for the pooled NHANES sample. The geometric means of TUA1 and TUA2 were 6.57 μg/L and 5.64 μg/L, respectively. After adjusting for age, sex, race, income, creatinine, and survey cycle, odds ratios for a negative VZV IgG result in association with 1-unit increases in natural log-transformed (ln)-TUA1 and ln-TUA2 were 1.87 (95% CI: 1.03, 3.44) and 1.40 (95% CI: 1.0, 1.97), respectively.

**Conclusions:**

In this cross-sectional analysis, urinary arsenic was inversely associated with VZV IgG seroprevalence in the U.S. population. This finding is in accordance with clinical observations of zoster virus reactivation from high doses of arsenic. Additional studies are needed to confirm the association and evaluate causal mechanisms.

**Citation:**

Cardenas A, Smit E, Houseman EA, Kerkvliet NI, Bethel JW, Kile ML. 2015. Arsenic exposure and prevalence of the varicella zoster virus in the United States: NHANES (2003–2004 and 2009–2010). Environ Health Perspect 123:590–596; http://dx.doi.org/10.1289/ehp.1408731

## Introduction

Initial exposure to the varicella zoster virus (VZV) causes chickenpox (varicella). Upon recovery from the initial infection, VZV establishes latency and if reactivated, it will cause shingles (herpes zoster; HZ) (Gershon et al. 2010). As long as the host maintains sufficient VZV-specific cell-mediated immunity, the virus can remain latent indefinitely. Approximately one of three people in the United States will develop HZ in their lifetime, resulting in > 1 million cases every year [Centers for Disease Control and Prevention (CDC) 2011a; Gershon et al. 2010]. The risk of HZ increases in the elderly and in people with immunosuppressive illnesses and/or those taking immunosuppressive medications (CDC 2011a). Other risk factors for HZ include diabetes, female sex, Caucasian race, medical trauma, and psychological stress (Gershon et al. 2010).

Interestingly, medical case reports dating back to the early 1900s have documented the appearance of fine punctate irritating rashes, herpetic skin eruptions, and HZ in people shortly after they recovered from acute and subchronic arsenic poisoning (Bartolomé et al. 1999; Carlill 1917; Hope-Simpson 1965; Jacob 1931; Satterlee 1960; Uede and Furukawa 2003; Walsh 1900). More recently, clinicians have identified HZ as a common side effect for patients with acute promyelocytic leukemia (APL) after they received treatment with arsenic trioxide (Au and Kwong 2005; Douer and Tallman 2005; Nouri et al. 2006; Rousselot et al. 2004; Subbarayan et al. 2007; Tanvetyanon and Nand 2004). Although no study has been conducted to determine whether HZ is more common among APL patients who receive arsenic trioxide versus other treatments, physicians have commented that only the patients treated with arsenic trioxide developed HZ (Tanvetyanon and Nand 2004). There is evidence from animal models and *in vitro* studies that arsenic exposure can alter the immune response (Dangleben et al. 2013). However, it is unknown whether exposure to arsenic from environmental sources affects the VZV immune response.

Currently, two National Health and Nutrition Examination Surveys (NHANES) included both VZV IgG serology testing and urinary arsenic measurements. Using these data, we sought to determine whether arsenic exposure in the general U.S. population is a risk factor for VZV IgG seronegativity. Our hypothesis was that higher urinary arsenic concentrations would be associated with a higher seronegative prevalence of VZV IgG.

## Methods

*Study population*. The National Health and Nutrition Examination Survey (NHANES) is designed to be a nationally representative sample of the resident, noninstitutionalized U.S. civilian population. Participation rates for the 2003–2004 and the 2009–2010 cycles were 76% and 77.3%, respectively (CDC 2013a). Informed consent was obtained from all survey participants, and all study protocols were approved by the National Center for Health Statistics research ethics review board (CDC 2013b).

In the present study, we restricted our analyses to individuals who had data on both VZV serology and urinary arsenic speciation. Serological testing for VZV antibodies was performed on participants 6–49 years of age. Urinary arsenic concentrations were measured in a random sample of one-third of the participants ≥ 6 years of age (Caldwell et al. 2009). This resulted in data on 1,641 individuals from the 2003–2004 survey and 1,718 individuals from the 2009–2010 survey. We excluded individuals with a positive test for human immunodeficiency virus (HIV; *n* = 4 for 2003–2004 and *n* = 7 for 2009–2010) due to their compromised immune status. This resulted in a final sample size of 3,348 for the two pooled survey cycles.

*Varicella zoster virus (VZV) serology*. Initially, NHANES utilized an enzyme immunoassay method that detects disease-acquired IgG antibodies to VZV but is less sensitive to the IgG response produced by the vaccine (Reynolds et al. 2010). Subsequently, NHANES adopted a glycoprotein-based (gp) enzyme-linked immunosorbent assay (ELISA) that is more sensitive and reliably detects both vaccine- and disease-induced immunity (Hammond et al. 2006). We utilized all available VZV data to ascertain seroprevalence status across survey cycles to identify 98 seronegative and 3,250 seropositive individuals who were used in the combined analysis.

NHANES 2003–2004. VZV IgG was measured in serum using a whole cell enzyme immunoassay (EIA). This assay was developed by the Immunoserology Unit of the California State Department of Health Services Viral and Rickettsial Disease Laboratory and has been previously described (Forghani et al. 1978). This protocol yields an index of optical density (OD) reading, where an OD > 1 indicates the presence of the VZV antibody (seropositive) and an OD < 1 indicates that the antibody was not detected (seronegative). A negative VZV IgG test is useful to determine whether an individual is susceptible to infection by the virus (CDC 2011b). Subsequently, the CDC retested a subsample from this survey cycle using a gp-ELISA, which is considered to have higher sensitivity and specificity compared to the EIA method, and determined that the EIA produced false-negative results for 26% of participants ages 6–19 years (Reynolds et al. 2010). NHANES made the gp-ELISA data available in 2009. Subsequently, we used data from both the EIA and the available gp-ELISA to classify VZV status in this cycle. Specifically, the EIA and the gp-ELISA methods agreed on 40 negative and 37 positive VZV individuals who were retested. Sixteen individuals who were initially identified as VZV negative by the EIA were reclassified as seropositive based on the gp-ELISA results. Finally, 14 individuals with an equivocal gp-ELISA result were left as initially identified by the EIA method. This resulted in 69 seronegative and 1,568 seropositive individuals for this cycle.

NHANES 2009–2010. In this survey the CDC evaluated the presence of the VZV antibody using an EIA as described above. However, all samples that tested negative (OD < 1) or in the equivocal range were reevaluated using the gp-ELISA method. Therefore, all negative VZV IgG results in this survey were confirmed, yielding greater accuracy for the serological classification. This resulted in 29 seronegative and 1,682 seropositive individuals in this cycle.

*Urinary arsenic assessment*. Urinary arsenic concentrations were measured in a spot sample collected during the physical examination and analyzed within 3 weeks of collection using high-performance liquid chromatography (HPLC) coupled to inductively coupled-plasma dynamic reaction cell–mass spectrometry (ICP-DRC-MS). Seven urinary arsenic species were evaluated with this method: arsenite (As^III^), arsenate (As^V^), arsenobetaine (AsB), arsenocholine (AsC), monomethylarsonic acid (MMA), dimethylarsenic acid (DMA), and trimethylarsine oxide, as well as total urinary arsenic (CDC 2007). The corresponding limits of detection (LODs) for the urinary arsenic species used were 0.4 μg/L, 0.6 μg/L, 1.2 μg/L, 1.0 μg/L, 0.9 μg/L, and 1.7 μg/L for AsB, AsC, As^III^, As^V^, MMA, and DMA, respectively. The LOD for total urinary arsenic in the 2003–2004 cycle was 0.6 μg/L and changed in the 2009–2010 cycle to 0.74 μg/L. Samples with arsenic measurements below the LOD were assigned a level equal to the LOD divided by the square root of two. The proportion of samples below the LOD in 2003–2004 was 44.57% for AsB, 98.9% for AsC, 93.28% for As^III^, 92.61% for As^V^, 60.26% for MMA, 11.46% for DMA, and 1.49% for total arsenic. In 2009–2010, the proportion of samples below the LOD was 37.14% for AsB, 98.2% for AsC, 94.97% for As^III^, 97.29% for As^V^, 67.39% for MMA, 19.93% for DMA, and 0.77% for total arsenic.

AsB and AsC are arsenosugars present in seafood and are considered to be nontoxic (Mürer et al. 1992). We defined total urinary arsenic (TUA) using two approaches. The first approach (TUA1) was defined as the sum of As^III^, As^V^, MMA, and DMA. The second approach (TUA2) was defined as total arsenic minus AsB and AsC. Because NHANES includes multiple demographic groups, it has been recommended that urinary analyte concentrations be evaluated as an unadjusted analyte concentration and have urinary creatinine as a separate independent variable to account for differences in urine dilution (Barr et al. 2005).

*Adjustment variables*. Variables that were considered in the analyses *a priori* as potential confounders for the association between VZV status and urinary arsenic concentrations included age, sex, race/ethnicity, family poverty–income ratio, body mass index (BMI), urinary creatinine levels, and survey cycle. Race/ethnicity was self-reported as non-Hispanic white, non-Hispanic black, other Hispanics, Mexican American, and other race including multiracial. “Other Hispanics” were collapsed into a single category with the other race category including multiracial for both survey cycles. BMI was calculated by dividing measured weight in kilograms by measured height in meters squared. BMI was classified as underweight (< 18.5), normal (18.5–24.9), overweight (25–29.9), and obese (≥ 30). For participants < 20 years of age, BMI classification was defined using the CDC growth charts for age- and sex-specific cutoffs. Urinary creatinine was right-skewed and subsequently natural log (ln)-transformed.

*Statistical analysis*. All statistical analyses were performed in Stata using the survey command to account for the complex sampling design (version 12.1; StataCorp LP). Unweighted sample sizes are presented along with weighted prevalence and geometric means (GMs) for each covariates. Standard errors (SEs) and confidence intervals (CIs) were estimated using the Taylor linearization method. The statistical significance level was set at α = 0.05, and all statistical tests were two-tailed. The association was evaluated for the two survey cycles independently. The two survey cycles were also combined to increase the precision for the estimated relationship, and survey weights were rescaled to match the U.S. population at midpoint for the combined survey cycles.

TUA concentrations (TUA1 and TUA2) were right-skewed and ln-transformed for the analyses. Linear regression models were used to calculate the GM and SE of TUA by the prevalence of VZV status, and all covariates were evaluated for both survey cycles and for the combined sample. Associations between all covariates and urinary arsenic were evaluated using a Wald test for significance to evaluate the overall association. The models estimating the GM of TUA for each strata always included urinary creatinine.

Logistic regression models were used to evaluate the association between seronegative VZV status and ln-TUA for the combined sample and for the two survey cycles independently. This approach included all *a priori* covariates that were considered risk factors for VZV (e.g., age, sex, race/ethnicity, family poverty–income ratio, BMI), NHANES survey year, and ln-urinary creatinine. For the combined sample, we tested the interaction between survey cycle and arsenic exposure in the adjusted model. These interactions were not significant for TUA1 (*p* = 0.68) and TUA2 (*p* = 0.39), which suggested that the associations did not differ by survey year and further supported combining the two survey cycles. As a sensitivity analysis we also analyzed these logistic regression models without the sample weights (unweighted). This unweighted approach would prevent spurious associations that could result if a heavy weight was attached to few individuals. Further, weighted penalized splines were used to evaluate nonlinear relationships with knots at the 5th, 25th, 50th, and 95th percentiles of exposure. Because weighted penalized splines using the pooled data may produce unreliable 95% CIs, we used generalized additive models to evaluate nonlinear relationships and compute 95% CIs without including survey weights.

## Results

Across both survey cycles, a total of 3,348 participants had TUA1 measurements and 3,283 participants had TUA2 measurements. The difference in sample size (*n* = 65) resulted from missing measurements used in computing TUA2. There were 98 individuals who were seronegative for the VZV IgG in the pooled study sample. For the pooled sample, the overall GM TUA1 and TUA2 were 6.57 μg/L (95% CI: 6.26, 7.91 μg/L) and 5.64 μg/L (95% CI: 5.20, 6.12 μg/L), respectively. The population characteristics for the pooled sample are provided in Table 1. TUA was significantly associated with race/ethnicity, age, and VZV serology based the overall creatinine-adjusted Wald test (Table 2). In the pooled sample, TUA1 and TUA2 were slightly higher among VZV-seronegative IgG participants compared with seropositive individuals (8.31 μg/L vs. 6.77 μg/L, *p* = 0.01; and 7.62 μg/L vs. 5.85 μg/L, *p* = 0.02). Only TUA1 was associated with BMI classification. The GMs of total urinary arsenic, which were adjusted for creatinine, were not different by sex or family poverty–income ratio (Table 2).

**Table 1:** Population characteristics [*n* (%)] for the combined NHANES 2003–2004 and 2009–2010 presented as unweighted sample sizes and weighted percentages.

Characteristic	TUA1	TUA2
Total sample size (*n*)	3,348	3,283
Sex
Male	1,673 (50.3)	1,649 (50.6)
Female	1,675 (49.7)	1,634 (49.4)
Race
Non-Hispanic white	1,215 (62.7)	1,177 (62.3)
Non-Hispanic black	845 (12.9)	839 (13.1)
Mexican American	868 (12.1)	853 (12.2)
Other/Other Hispanic	420 (12.3)	414 (12.4)
Family poverty–income ratio
≤ 1 (below poverty level)	943 (19.6)	926 (80.2)
> 1 (above poverty level)	2,185 (81.4)	2,139 (19.8)
Missing	220	218
BMI (kg/m^2^)
< 18.5 (underweight)	86 (2.2)	85 (2.2)
18.5–24.9 (normal)	1,498 (41.2)	1,469 (41.0)
25–29.9 (overweight)	825 (28.0)	813 (28.1)
≥ 30 (obese)	909 (28.6)	916 (28.7)
Missing	30	23
Age (years)
6–11	514 (11.5)	534 (11.1)
12–19	1,002 (17.6)	1,063 (17.8)
≥ 20	1,598 (70.9)	1,686 (71.1)
TUA1 (μg/L)
≤ 4.8 (tertile 1)	1,032 (33.7)	—
> 4.8 to 7.5 (tertile 2)	1,116 (32.3)	—
> 7.5 to 139 (tertile 3)	1,200 (34.0)	—
TUA2 (μg/L)
≤ 3.7 tertile 1	—	1,004 (33.0)
> 3.7 to 8.4 (tertile 2)	—	1,132 (33.2)
> 8.4 to 300 (tertile 3)	—	1,147 (33.8)
Creatinine (mg/dL)
≤ 80 (tertile 1)	960 (31.6)	917 (30.4)
> 80 to 153 (tertile 2)	1,122 (32.6)	1,113 (33.1)
> 153 to 768 (tertile 3)	1,263 (35.8)	1,253 (36.5)
VZV IgG
Seropositive	3,250 (97.8)	3,187 (97.8)
Seronegative	98 (2.2)	96 (2.9)
TUA1 = As^III^ + As^V^ + MMA + DMA. TUA2 = Total As – AsC – AsB.		

**Table 2:** Weighted geometric mean (GM) and SE of total urinary arsenic (μg/L) by demographic characteristics in the NHANES 2003–2004 cycle, 2009–2010 cycle, and a pooled sample (2003–2004 and 2009–2010).

Characteristic	2003–2004				2009–2010				Pooled			
	TUA1 GM (SE)	TUA1 *p*‑Value^*a*^	TUA2 GM (SE)	TUA2 *p*‑Value^*a*^	TUA1 GM (SE)	TUA1 *p*‑Value^*a*^	TUA2 GM (SE)	TUA2 *p*‑Value^*a*^	TUA1 GM (SE)	TUA1 *p*‑Value^*a*^	TUA2 GM (SE)	TUA2 *p*‑Value^*a*^
Seronegative (*n*)	69		69		29		29		98		98
Sex		0.27		0.94		0.09		0.01		0.58		0.15
Male	6.91 (1.05)		5.80 (1.09)		6.30 (1.03)		5.33 (1.04)		6.67 (1.03)		5.64 (1.05)
Female	6.96 (1.03)		5.83 (1.08)		6.68 (1.04)		5.94 (1.05)		6.77 (1.03)		5.95 (1.05)
Race		< 0.001		< 0.001		< 0.001		< 0.001		< 0.001		< 0.001
Non-Hispanic white	6.42 (1.04)		5.23 (1.09)		5.92 (1.03)		5.28 (1.04)		6.23 (1.03)		5.22 (1.06)
Non-Hispanic black	6.52 (1.04)		5.63 (1.07)		6.00 (1.04)		5.22 (1.07)		6.31 (1.03)		5.40 (1.05)
Mexican American	7.89 (1.05)		6.85 (1.05)		7.19 (1.03)		5.77 (1.05)		7.34 (1.03)		6.25 (1.03)
Other/Other Hispanic	10.79 (1.06)		11.76 (1.10)		10.66 (1.10)		10.42 (1.08)		10.22 (1.05)		11.06 (1.07)
Family poverty–income ratio		0.69		0.95		0.54		0.15		0.53		0.39
≤ 1 (below poverty level)	6.87 (1.03)		5.84 (1.08)		6.40 (1.03)		5.84 (1.04)		6.69 (1.02)		5.81 (1.05)
> 1 (above poverty level)	7.00 (1.07)		5.87 (1.10)		6.55 (1.04)		5.34 (1.06)		6.82 (1.04)		5.60 (1.06)
BMI (kg/m^2^)		0.002		0.36		0.70		0.05		0.03		0.19
< 18.5 (underweight)	7.16 (1.09)		6.11 (1.08)		6.41 (1.16)		6.44 (1.15)		6.99 (1.06)		5.96 (1.04)
18.5–24.9 (normal)	7.17 (1.04)		6.11 (1.08)		6.52 (1.03)		5.82 (1.06)		6.85 (1.03)		6.97 (1.04)
25–29.9 (overweight)	6.93 (1.05)		5.84 (1.09)		6.56 (1.04)		6.20 (1.04)		6.86 (1.04)		5.94 (1.05)
≥ 30 (obese)	6.46 (1.03)		5.51 (1.07)		6.33 (1.03)		5.50 (1.04)		6.43 (1.02)		5.50 (1.04)
Age (years)		0.002		< 0.001		< 0.001		< 0.001		< 0.001		< 0.001
6–11	7.51 (1.06)		6.78 (1.11)		7.25 (1.03)		5.87 (1.06)		7.27 (1.04)		6.26 (1.06)
12–19	6.41 (1.05)		4.66 (1.07)		5.60 (1.03)		3.96 (1.05)		5.90 (1.03)		4.29 (1.04)
≥ 20	7.27 (1.04)		5.93 (1.08)		6.62 (1.03)		6.15 (1.04)		6.82 (1.03)		6.03 (1.04)
VZV IgG		0.08		0.06		0.004		0.02		0.01		0.02
Seropositive	7.09 (1.04)		6.16 (1.07)		6.47 (1.03)		5.61 (1.04)		6.77 (1.02)		5.85 (1.04)
Seronegative	8.61 (1.12)		8.21 (1.17)		8.18 (1.07)		7.62 (1.13)		8.31 (1.08)		7.62 (1.12)
TUA1 = As^III^ + As^V^ + MMA + DMA. TUA2 = Total As – AsC – AsB. ^***a***^Models adjusted for ln-transformed creatinine.												

In adjusted models for the pooled survey sample, odds ratios (aORs) for a negative VZV IgG result in association with 1-unit increases in ln-TUA1 and ln-TUA2 were 1.87 (95% CI: 1.03, 3.44) and 1.40 (95% CI: 1.0, 1.97), respectively (Table 3). These models were adjusted for age, ln-urinary creatinine, sex, race/ethnicity, family poverty–income ratio, BMI classification, and survey cycle. Because of the relatively small sample size and because some data were missing, we also analyzed the association between arsenic and VZV status using a more parsimonious approach that only controlled for age, survey cycle, and urinary creatinine. These models without as much missing data yielded consistent results where the odds of having a negative VZV result increased with each increase in ln-TUA1 (aOR = 2.24; 95% CI: 1.37, 3.56) and ln-TUA2 (aOR = 1.58; 95% CI: 1.17, 2.13). Furthermore, the unweighted adjusted models yielded consistent results, suggesting that the observed association was not driven by spurious data that can result if a few individuals are given a greater weight. Finally, additional analysis that examined the association between TUA and VZV seroprevalence in each survey cycle independently yielded a consistent association between negative VZV IgG seroprevalence and arsenic (Table 3).

**Table 3:** Adjusted odds ratios (aORs) and 95% CIs for seronegative VZV status in association with a 1-unit increase in ln-transformed TUA (μg/L) for the combined NHANES sample (2003–2004 and 2009–2010).

	2003–2004		2009–2010		Pooled	
	Weighted^*a*^ aOR (95% CI)	Unweighted^*a*^ aOR (95% CI)	Weighted^*a*^ aOR (95% CI)	Unweighted^*a*^ aOR (95% CI)	Weighted^*b*^ aOR (95% CI)	Unweighted^*b*^ aOR (95% CI)
ln-TUA1	1.87 (1.05, 3.36)	2.03 (1.22, 3.37)	2.29 (1.49, 3.51)	1.92 (1.00, 3.71)	1.87 (1.03, 3.44)	1.95 (1.32, 2.90)
ln-TUA2	1.26 (0.97, 1.66)	1.36 (0.99, 1.89)	1.67 (1.17, 2.40)	1.44 (0.89, 2.33)	1.40 (1.00, 1.97)	1.37 (1.04, 1.79)
TUA1 = As^III^ + As^V^ + MMA + DMA. TUA2 = Total As – AsB – AsC. ^***a***^Models adjusted for age, sex, race, family poverty–income ratio, BMI classification, and ln urinary creatinine. ^***b***^Models adjusted for age, sex, race, family poverty–income ratio, BMI classification, ln urinary creatinine, and survey cycle.						

**Figure 1:**
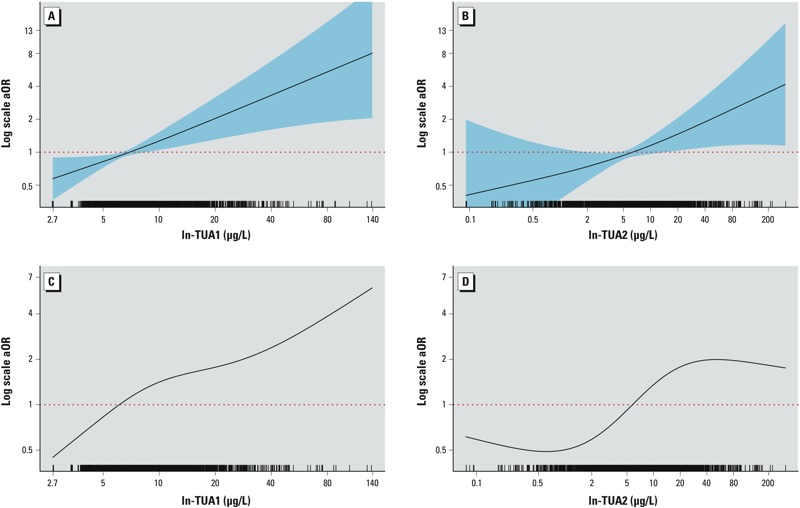
Adjusted odds ratios (aORs) from penalized spline models for negative VZV IgG by TUA. (*A*) TUA1 unweighted. (*B*) TUA2 unweighted. (*C*) TUA1 weighted. (*D*) TUA2 weighted. aORs are based on penalized splines for ln-transformed total arsenic exposure. Models fully adjusted for age, ln-urinary creatinine, sex, race/ethnicity, family poverty–income ratio, BMI classification, and survey cycle. (*A*) and (*B*) are unrestricted splines with 95% CIs; (*C*) and (*D*) were estimated from weighted models restricted to knots at the 5th, 25th, 50th, and 95th percentiles of exposure. TUA1 = As^III^ + As^V^ + MMA + DMA. TUA2 = Total As – AsC – AsB.

The shape of the dose–response relationship between TUA and VZV seronegative status was modeled using penalized splines as both weighted and unweighted samples (Figure 1). The shape of the dose–response relationships was approximately linear for unweighted spline models of TUA1 and TUA2 (Figure 1A and 1B, respectively). The weighted dose–response curves were more attenuated and nonlinear for TUA1 and TUA2 (Figure 1C and 1D, respectively). Despite these subtle differences in the shape of the dose–response curve between arsenic and VZV seronegative status, the weighted and unweighted analyses yielded a consistent positive association.

## Discussion

In a representative sample of the U.S. population 6–49 years of age, higher concentrations of TUA were associated with a higher prevalence of negative VZV serology results after adjusting for other risk factors. Although the shape of the dose–response relationship could have been biased by accounting for urinary metabolites that were below the limit of detection, a significant positive association was observed in two NHANES surveys taken 6 years apart, where the odds of a negative VZV result increased approximately 40–95% for each unit increase in TUA1 or TUA2. The association was consistent in the pooled analytic sample and across each survey cycle. These findings, which are the first to look at the association between arsenic exposures and VZV status in the U.S. general population and across a relatively modest range of exposures, build upon prior experimental evidence and clinical observations that showed that therapeutic doses of inorganic arsenic affect and/or suppress specific immune functions; our findings also support the hypothesis that arsenic exposure diminishes VZV immunity.

Arsenic is a common environmental contaminant that can be found in groundwater and in the food chain. In our study, it was not possible to determine the route of arsenic exposure or the species of arsenic that comprised the exposure because only a spot urine sample was collected for arsenic measurement. However, in the United States, arsenic-contaminated drinking water is considered to be the dominant source of exposure and is mostly a concern for communities that rely on groundwater as their source of potable water [U.S. Environmental Protection Agency (EPA) 2001]. A 2001 study that used data collected by the U.S. EPA estimated that 34 million Americans were drinking water with average arsenic concentrations > 50 μg/L, which was the maximum contaminant level (MCL) for drinking water at that time (Mushak 2000). In 2001, the U.S. EPA lowered the arsenic MCL to 10 μg/L because of concerns about elevated risk of internal cancers; municipalities had until 2006 to comply (Abedin et al. 2002). Private drinking-water wells, however, are not monitored or regulated by the U.S. EPA, and survey data suggests that 11–19% of private wells exceed 10 μg As/L (Focazio et al. 2006; Montgomery et al. 2003; Twarakavi and Kaluarachchi 2006). Crops, particularly rice and cereal grains, can also take up arsenic from the soil and irrigation water (Cascio et al. 2011), and dietary sources of arsenic are receiving more attention as a result of data from recent studies indicating that people who regularly consume rice have higher urinary arsenic levels (Davis et al. 2012). It is possible that the lower exposure levels measured in the present study resulted from dietary intake. Although we were able to calculate a TUA concentration that did not include nontoxic arsenosugars, the small sample size prohibited us from examining the association between VZV status and individual urinary arsenic metabolites.

There have been many clinical reports of VZV reactivation among people who have survived acute arsenic poisoning and among patients with acute promyleotic leukemia who have been treated with arsenic trioxide (Au and Kwong 2005; Bartolomé et al. 1999; Douer and Tallman 2005; Hope-Simpson 1965; Nouri et al. 2006; Rousselot et al. 2004; Subbarayan et al. 2007; Tanvetyanon and Nand 2004). Yet the biological mechanisms by which this occurs are not well understood. Researchers have shown that arsenic trioxide prevents the loss of virions from the perinuclear cell region, leading to an increase in cellular vector genome retention (Mitchell et al. 2013). There is also considerable evidence from *in vitro* and *in vivo* experimental studies that inorganic arsenicals are potent immunotoxicants. High doses of inorganic arsenic are known to suppress IgM and IgG antibody formation; inhibit antigen-driven T-cell proliferation and macrophage activity; block the differentiation of monocytes into functional macrophages; decrease CD4+ splenic cell numbers; and alter the development, activation, and proliferation of T cells (Bourdonnay et al. 2009; Burchiel et al. 2009; Burns and Munson 1993; Conde et al. 2007; Dangleben et al. 2013; de la Fuente et al. 2002; Galicia et al. 2003; Hernández-Castro et al. 2009; Kozul et al. 2009a; Lemarie et al. 2006a, 2006b; Martin-Chouly et al. 2011; Nain and Smits 2012; Patterson et al. 2004; Sikorski et al. 1989; Soto-Peña et al. 2006; Yoshida et al. 1987). In addition, data from experimental models show that high doses of inorganic arsenic influence viral pathology (Dangleben et al. 2013; Kozul et al. 2009b; Martin-Chouly et al. 2011; Mitchell et al. 2013; Patterson et al. 2004; Ramsey et al. 2013; Sebastian et al. 2006). Less is known about the effects of methylated arsenical species on immunological outcomes.

The best way to reduce the risk of chicken pox and HZ is to be vaccinated for VZV. Since 1996, the Advisory Committee on Immunization Practices has recommended routine VZV vaccination of all children 18 months to 2 years of age, susceptible adolescents, and adults that are at high risk of exposure to the virus (Marin et al. 2007). The vaccine contains live attenuated VZV, which is very effective and induces immunity in > 95% of the people who receive it (Reynolds et al. 2010). The vaccine results in latent infection, which can be reactivated and cause HZ, although studies have shown that the risk of reactivation after the vaccine is lower than for people who were infected with wild-type VZV (Reynolds et al. 2010). It would be useful for future studies to consider whether the arsenic–VZV seroprevalence association is only in those with wild-type VZV compared with people who have been vaccinated against VZV.

Important strengths of the present study include the use of a representative sample of the U.S. population exposed to arsenic at environmental concentrations. We also used urinary biomarkers to assign personal exposure levels of arsenic. Further we adjusted for relevant risk factors for VZV and urinary arsenic concentrations. The rigorous quality control procedures implemented in NHANES is also an important strength of the quality of the data presented. Although the protocol for the VZV assay changed between cycles, there was improved precision in the data collected in 2009–2010, which is likely a function of the gp-ELISA for determining VZV status. This assay has a higher sensitivity and specificity for VZV antibodies produced by vaccination, which likely accounts for the more precise ORs in this cycle, even though the actual number of VZV-seronegative samples was lower. Finally, the reproducibility of the effect and the observation of an exposure–response relationship between increasing TUA concentration and VZV serology in two different survey cycles is reassuring.

There are also limitations to our study that must be considered. NHANES is a cross-sectional study, and the temporality between arsenic exposure and VZV serology cannot be assessed. NHANES measures urinary arsenic concentrations only in one-third of the participants, and urinary arsenic metabolites have a relatively short half-life, which limits their use for estimating historical or long-term exposures (Buchet et al. 1981). The presence of unmeasured confounders cannot be ruled out because serum-specific IgG response resulting from vaccination or natural infection could be modified by other immune-suppressive conditions or chronic infections. Yet, we accounted for several important risk factors in the population, including age, race, sex, and BMI. Also, the seronegative VZV prevalence was low in both cycles for our combined sample. It is possible that participants who were infected with VZV previously but did not mount a sufficient IgG response that could allow for accurate serology testing could have been misclassified. Furthermore, a negative VZV IgG result could indicate that an individual has not been previously infected with the virus and that arsenic exposure was protective. This interpretation, however, seems unlikely because arsenic is known to be immunotoxic, clinical reports have noted VZV reactivation after arsenic exposure, an estimated 99.5% of people born in the United States who are ≥ 40 years of age have serological evidence of previous VZV, and VZV vaccines were widely adopted after 1996 (CDC 2012). Unfortunately, there was no available information on other antibodies, such as IgM, to improve our classification of VZV status. We were also not able to evaluate the observed association in children < 6 years of age or adults > 45 years of age. VZV reactivation is a common problem among older adults, with the incidence of zoster increasing at 50–60 years, so the impact of arsenic exposure on this group may be different (Harpaz et al. 2008).

## Conclusions

TUA concentration was positively associated with seronegative VZV IgG prevalence in a population with modest arsenic exposure. This information builds upon experimental studies and clinical observations showing an association between acute exposures to high levels of arsenic with herpes zoster and supports a link between environmentally relevant levels of arsenic exposure and VZV-specific immune response. Additional studies are needed to more fully evaluate the effect of arsenic on other parameters of immune functioning and its ability to cause VZV reactivation. From a public health perspective, confirmation of arsenic’s ability to suppress specific immune functioning has important implications for vaccine-preventable illnesses.
